# Neurobehavioral and Neuropathological Alterations Induced by Nickel Sulphate Toxicity in Rats: Molecular Mechanisms and Prophylaxis with Curcumin Supplementation

**DOI:** 10.1007/s12011-025-04654-6

**Published:** 2025-06-18

**Authors:** Sally Mehanna, Neven H. Hassan, Marwa A. Ibrahim, Faten F. Mohammed, Eman I. Hassanen

**Affiliations:** 1 Department of Biotechnology, Faculty of Nanotechnology for Postgraduate Studies, Cairo University, Cairo, Egypt; 2 Department of Physiology, Faculty of Veterinary Medicine, Cairo University, Giza, 12211 Egypt; 3 Department of Biochemistry, Faculty of Veterinary Medicine, Cairo University, Giza, 12211 Egypt; 4 Department of Pathology, Faculty of Veterinary Medicine, Cairo University, Giza, 12211 Egypt; 5 Department of Pathology, College of Veterinary Medicine, King Faisal University, Al Ahsa, 31982 Saudi Arabia

**Keywords:** Curcumin, Neuropathology, Neurotoxicity, Nickel sulfate, Gene regulation

## Abstract

Nickel is recognized as an environmental contaminant and can affect various living organisms; therefore, clarifying the mechanism of Ni-induced toxicity and elucidating a protection strategy are crucial. This study investigated the neurotoxic potential of dosing nickel sulfate (Ni) on rats and the possible protective mechanism of curcumin in alleviating brain injury. Twenty-eight male albino Wistar rats were allocated into four groups: G1; control, G2; curcumin (CUR, 1.5 mg/kg bwt/day, i.p), G3; nickel sulfate (Ni, 20 mg/kg bwt, per os), and G4; Ni + CUR by the same protocol dosing for 30 days. Results revealed that Ni induced neurobehavioral abnormalities including disruption in cognition, impaired memory, increased anxiety, and motor imbalance, impaired acetylcholinesterase expression, and disrupted brain redox state. Downregulation of Nrf2 and HO-1 expression was recorded; moreover, marked neuropathological alterations affect many brain areas, mainly the cerebrum, hippocampus, and cerebellum, with increased immune reactivity of caspase-3 and NF-κB. However, curcumin significantly reduces brain injury via down-regulation of the redox state and regresses the related neurobehavioral and neuropathological alterations. The role of curcumin in the mitigation of Ni-induced intoxication was confirmed via antioxidant and anti-apoptotic pathways and downregulation of NF-κB in the brain.

## Introduction

Rapid advancements in technology and industries have caused an increment in environmental problems. Consequently, heavy metal deposits have increased in air, land, and water [1, 2]. Among these heavy metals, nickel, an important component of over a hundred compounds, requires special attention due to its expanding industrial application and potential toxic properties [3–5]. As a result of its widespread industrial use, nickel has been polluting the environment during its production, recycling, and disposal. Various industries release nickel into the atmosphere, including nickel–cadmium battery industries, trash incinerators, oil-burned power plants, coal-burned power plants, plastic and rubber industries, and electroplating industries [1].

In case of occupational or environmental exposure to nickel compounds, the brain, lungs, gonads, skin, kidneys, and liver are among the organs that can be adversely affected. A particular target of Ni is the nervous system, leading to several neurological signs [6, 7]. Additionally, nickel is also known to have significant effects on the cardiovascular, reproductive, and neurological systems during short-term exposure. Exposure to nickel induces the formation of free radicals in a variety of tissues in humans as well as animals, which in turn increases lipid peroxidation [8]. Nuclear factor-2 erythroid-2 (Nrf-2) plays a crucial role in combating ROS and enhancing other intrinsic genes, including hemeoxygenase-1 (HO-1), which eliminates ROS and LPO and protects against neurodegeneration brought on by oxidative stress [9]. Additionally, neuronal oxidative stress has been shown to trigger transcription factors and proinflammatory mediators such as NF-κB, leading to neuroinflammation and neurodegeneration [10]. According to research conducted on laboratory animals and nickel workers, the US Department of Health and Human Services and the International Agency for Research on Cancer (IARC) have designated all nickel compounds aside from metallic nickel as human carcinogens. Nevertheless, research on the neurotoxic consequences of nickel is limited, reflecting environmental health issues related to nickel.

Recent epidemiological studies suggest that the onset of neurodegenerative diseases may be postponed by natural antioxidant agents, including polyphenols, fatty acids, and vitamin-rich foods [11, 12]. Accordingly, there has been a surge in attention to the application of phytochemicals in the pharmaceutical drug industry, particularly in the context of the potential of a natural compound like curcumin as an appropriate replacement strategy for the treatment of heavy metal-induced toxicity [13, 14]. Curcumin (diferuloylmethane) is a hydrophobic polyphenol that is derived from the rhizomes of the Curcuma Longa Linn plant, which is a member of the Zingiberaceae family and is often known as turmeric. Curcumin has been traditionally employed as a treatment for a variety of diseases in Asian nations [15, 16].

Curcumin possesses a diverse range of biological and pharmacological effects, such as neuroprotective characteristics, anti-atherosclerotic, anti-inflammatory, and antioxidant agent, attributable to its capacity to alter several signaling molecules [17, 18]. Additionally, it prevents renal toxicity and hepatic injury; inhibits cataract, and gallstone development; facilitates muscle regeneration and wound healing; and provides therapeutic effects against diabetes, multiple sclerosis, and psoriasis [19]. Lately, curcumin has drawn attention as a neuroprotective agent against many toxicants and drugs, including aluminum, cobalt, and cyclophosphamide [20–22] Considering the aforementioned knowledge, the current work aims to assess the possible detrimental effects of nickel sulphate on various brain regions in rodents to comprehend the potential neuronal risk associated with human exposure and to determine the precise mechanistic molecular pathway that is responsible for mitigating these adverse effects by administering curcumin. This investigation differs from previous studies in two aspects. The first is that this manuscript comprehensively explained the pathway of Ni-induced neurotoxicity in rats, emphasizing the role of Nrf-2, HO-1, NF-κB, and caspase-3. Second, a novel method of administering curcumin—intraperitoneal injection—was employed to reduce such toxicity at extremely low dosage level (1.5 mg/kg bwt, 3 times a week). We predict that even at extremely low doses, intraperitoneal injection of curcumin can improve its bioavailability and efficacy more than those taken orally.

## Materials and Methods

### Chemicals

Nickel sulphate hexahydrate 97% (NiSO_4_.6H_2_O), CAS NO. [10101–97-0] purchased from CHEMBIO Co., PVT. Ltd., Navi-Mumbai, India. Curcumin was acquired from Sigma-Aldrich, USA. It was dissolved in dimethoxy sulfoxide (DMSO) and diluted with sterile normal saline according to the required dose.

### Animal Care and Treatments

Twenty-eight adult male rats of the Wistar strain were purchased from VACSERA animal husbandry (Helwan, Egypt) with a weight of 150 ± 10 g. Rats were kept in plastic cages and supplied commercial pellets (Al-Watania Com. for Fodder, El-Dakahleya, Egypt) and water ad libitum. Acclimatization of rats and monitoring were performed. All the procedures were done following the ARRIVE guidelines (PLoS Bio 8(6), e1000412,2010) in accordance with UK Guidance on the operation of the Animals (Scientific Procedures) Act 1986, and the Institutional Animal Care Committee of Cairo University (IACUC) has approved the experimental protocol (Approval number: Vet CU 25122023865).

The experimental groups were designed as follows: G1: the control group that was orally received normal saline; G2: intraperitoneally injected with curcumin (CUR, 1.5 mg/kg bwt, 3 times a week); G3: received nickel sulfate PO (Ni, 20 mg/kg bwt representing 1/10 LD50, every day); and G4: received both Ni and CUR by the same protocol dosing for 30 days. The number of rats was seven per group, while the treatment doses for Ni and curcumin were assigned according to previous studies [23, 24].

### Behavioral Assay

#### Dark Light Transition Test

One of the most basic and well-known tests for assessing anxiety and emotionality is the light–dark test. The device consisted of an open-topped, lighting partition joined to a closed-topped, darkened partition, with each partition measuring 30 × 40 × 40 cm. The partitions are connected by a small opening, which allows the rat to navigate between them. The rat was placed in a lighting partition and freely moved between the partitions for 5 min, and its position was monitored. The time spent using the lighting side against the darkened side during the 5-min assessment was monitored and used as an indicator of anti-anxiety behavior [25].

#### Rod Walking Test

Rats’ tendency to maintain their equilibrium on immovable horizontal rods is used to assess their psychomotor coordination. The rats were placed at the center of a horizontal elevating rod (100 cm long, 2.6 cm in diameter). The period that it took the rat to fall into a cushion placed underneath was timed for 6 s [25].

#### Spatial Y-Maze Test

The Y-maze test is used in rats to assess spatial working memory as well as locomotion. The Y-maze device has three arms designated A, B, and C that measure 30 cm in height, 15 cm in width, and 40 cm in length. Individual rats were placed at one of the arm’s ends and given 5 min to move randomly through the maze. The arm entrance sequence (i.e., ACBABABCBCBA) was recorded, and the percentage of spontaneous alternation behavior was calculated by dividing the number of actual alternations by the total number of possible alternations [26].

### Sampling

After 30 days, all rats were euthanized by cervical dislocation, and brain specimens were collected. Part of them were fixed in 10% neutral buffer formalin, while others were preserved at − 80c till used for biochemical and molecular analysis.

### Oxidative Stress and Choline Esterase (CHE) Determination

Brain tissue homogenate was used. Malondialdehyde (MDA) was evaluated according to Ohkawa et al. [27], reduced glutathione (GSH) levels were determined following Katerji et al. [28], nitric oxide (NO) was estimated as previously mentioned [29], and choline esterase activity was evaluated according to the modified Ellman method [30]. All kits used were purchased from Bio-diagnostic company, Egypt.

### Molecular Assay

#### RNA Isolation and Quantitative Real-Time PCR Analysis for Target Genes

Following the instructions provided by the manufacturer, the RNeasy Mini Kit (Qiagen Cat No./ID: 74104) was used to extract the total RNA from the brain tissue. We evaluated the isolated RNA’s content and purity at an optical density of A260/A280 = 1.9–2 [31]. SuperScript Reverse Transcriptase (ThermoFisher Scientific) was used to create the cDNA synthesis. As directed by the manufacturer, quantitative real-time PCR was performed using the ABI Prism StepOnePlus Real-Time PCR System (Applied Biosystems) and SYBR™ Green PCR Master Mix (ThermoScientific Cat number: 4309155) [32]. Table 1 contains a list of the target gene primer sets. The qRT-PCR was adjusted to the following program: an initial denaturation of 10 min at 95 °C followed by 40 cycles of 20 s/95 °C, and 30 s/60 °C. By gradually increasing the temperature from 65 to 90 °C, the melting curves for each reaction were generated [33]. The assay was created with duplicates of each sample and a no template control [34]. Using the normalized CT method, the expression values were normalized using the ACTB as an internal control [35]

**Table 1:** The primer sequence of the target genes

	Sense	Antisense	Amplicon	Accession no
*Nrf-2*	TGTAGATGACCATGAGTCGC	TCCTGCCAAACTTGCTCCAT	159	NM_031789.2
*HO-1*	AGCGAAACAAGCAGAACCCA	ACCTCGTGGAGACGCTTTAC	166	**NM_012580.2**
*ACTB*	CCGCGAGTACAACCTTCTTG	CAGTTGGTGACAATGCCGTG	297	NM_031144.3

### Histopathological Assay

Brain tissue specimens were collected and fixed in buffered neutral formalin, and routinely processed, paraffin embedded, and stained with H&E [36]. Tissue sections were examined in an Olympus BX43 light microscope and captured using an Olympus DP27 camera linked to the cellSens dimensions software (Olympus).

### Immunohistochemical Assay

Unstained paraffin sections loaded on positively charged slides were further processed by immunohistochemistry technique to evaluate immunoreactivity against Caspase-3 and NF-κB detection of apoptosis and oxidative stress in brain tissue. Briefly, tissue sections were cleared in xylene for 15 min followed by rehydrating in decreasing grades of alcohol, washed with phosphate buffer saline (PBS) at pH 7.4 for 5 min. Antigen retrieval procedures were performed in the microwave after immersion in Tris/EDTA buffer (pH 9.0) for 5 min. Incubation with H2O2 for 5 min for blocking of endogenous peroxidase and followed by the addition of bovine serum for 30 min, incubation with primary anti-Caspase-3 antibody (dilution,1:100,ab224271, Abcam) and NF-kB (dilution,1:100,ab32536, Abcam) for 1 h, washing with PBS was carried out followed by incubation with HRP labeled secondary antibody and DAB-substrate kit (Bio SB, USA), and counter stain with Mayer’s hematoxylin then was applied.

### Statistical Analysis

SPSS Inc., Chicago, IL, USA, version 16.0, was used for statistical analysis. Means ± SEM was used to express the data. One-way analysis of variance (ANOVA) was used to compare the means (ANOVA), whereas the LSD post hoc test was utilized to evaluate mean differences across multiple comparisons. *P* ≤ 0.05 was the threshold for statistical significance.

## Results

### Behavioral Assessment

#### Light–Dark Transition Test

Rats exposed to Ni significantly reduced their period spent in the light partition while significantly increasing their time spent in the dark partition as compared to rats in all other groups. On the other hand, rats co-administered Ni with CUR significantly increase the duration spent in the light partition and decrease the duration spent in the dark partition compared to the Ni group (Table 2).

**Table 2:** Effect of nickel sulphate and/or curcumin on some neurobehavioral parameters

Parameters/groups	Control	Ni	CUR	Ni + CUR
Light/dark transition test				
Time spent in the light compartment (s)	289.4 ± 3.58a	11.2 ± 4.68b	289.6 ± 3.20a	69.8 ± 3.54c
Time spent in the dark compartment (s)	10.6 ± 3.58a	288.8 ± 4.68b	10.4 ± 3.20a	230.2 ± 3.54c
Rod walking test				
Latency to fall	58.4 ± 0.81a	3.4 ± 0.50b	59.2 ± 0.37a	57.8 ± 0.37a
Spatial Y-maze test				
Spontaneous alternation behavior %	90.6 ± 4.16a	5.6 ± 5.6b	91.6 ± 3.61a	79.2 ± 4.42a

Data are expressed as the mean ± SEM (*n* = 7 rats/group). Different letters indicate statistically significant differences at *P* ≤ 0.05

#### Rod Walking Test

The Ni group showed a significant reduction in falling time in comparison with the other groups. However, the effects of Ni were alleviated by the coadministration of CUR, as there was a non-significant difference in falling times compared to the control rats (Table 2).

#### Spatial Y-Maze Test

The rats exposed to Ni showed a significant impairment in spatial working memory in the Y-maze device, as demonstrated by a reduction in spontaneous alternation behavior compared with rats in other groups. Conversely, Ni rats co-administered with CUR produced a significant enhancement of spatial cognition abilities compared with those administered Ni indicated by an increment in spontaneous alternation behavior (Table 2).

### Oxidative Stress and Choline Esterase (CHE) Determination

Data presented in Table 3 revealed a significant elevation of MDA and NO with a reduction in GSH levels and choline esterase activity in the brain tissue of the group that received Ni when compared to the control group. On the other hand, the group treated with CUR revealed significant restoration of GSH levels besides choline esterase activity and decrease of MDA as well as NO levels in the brain tissue when compared to the group that received Ni only (*P* ≤ 0.05).

**Table 3:** Effect of CUR and/or Ni on brain redox status and CHE activity

	CHE (U/L)	MDA (nmol/g tissue)	GSH (mg/g tissue)	NO (µmol/L)
Control	5515.75 ± 35.86 a	28.99 ± 2.38 a	45.42 ± 1.73 a	17.54 ± 1.70 a
CUR	5571.71 ± 43.11 a	27.32 ± 1.93 a	47.04 ± 2.45 a	19.22 ± 1.91 a
Ni	5079.51 ± 39.44 b	57.81 ± 3.66 b	28.50 ± 1.43 b	39.062 ± 1.84 b
Ni + CUR	5359.27 ± 33.87 c	42.34 ± 1.93 c	41.25 ± 1.75 a	30.01 ± 1.32

Data expressed as mean ± SEM (*n* = 7 rats/group). Different subscribe letters (a, b, c) show significant difference between groups at *P* ≤ 0.05

### RNA Isolation and Quantitative Real-Time PCR Assay

**Fig. 1:**
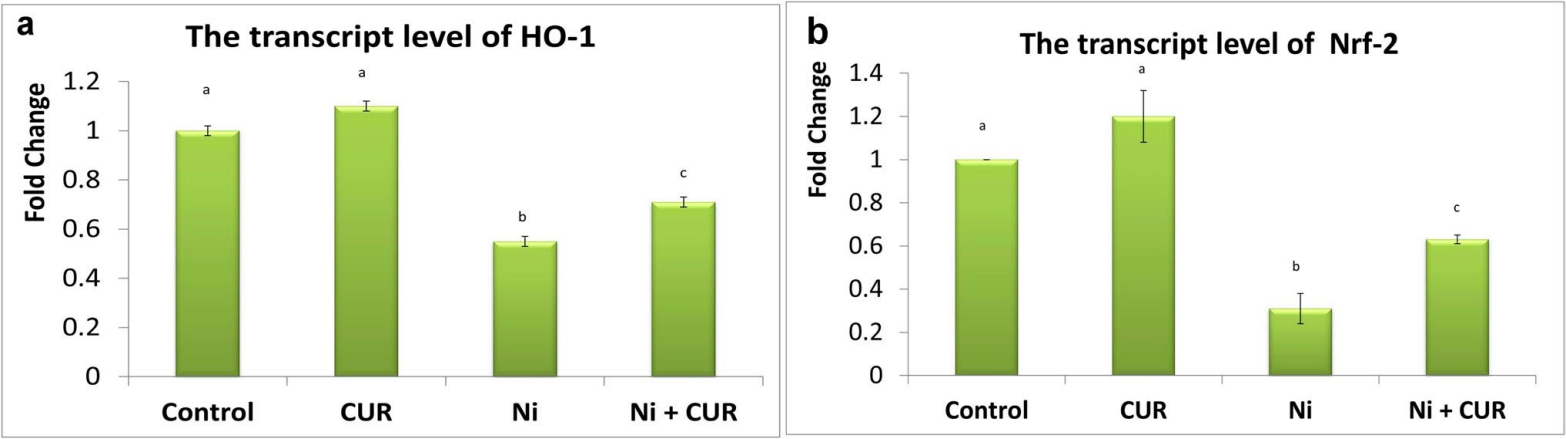
Bar charts representing the transcript level of HO-1 (**a**) and Nrf-2 (**b**) in the brain of various experimental groups. Data expressed as mean ± SEM (*n* = 5); different subscribe letters (a, b, c) show significant difference between groups at *P* ≤ 0.05

Data presented in Fig. 1 revealed a significant reduction in the transcriptase levels of *Nrf2* and *HO-1* genes in the brain tissue of the group that received Ni when compared to the control group. On the other hand, the group treated with CUR revealed a significant restoration of both gene levels in the brain tissue when compared to the group that received Ni only (*P* ≤ 0.05).

### Histopathological Analysis

**Fig. 2:**
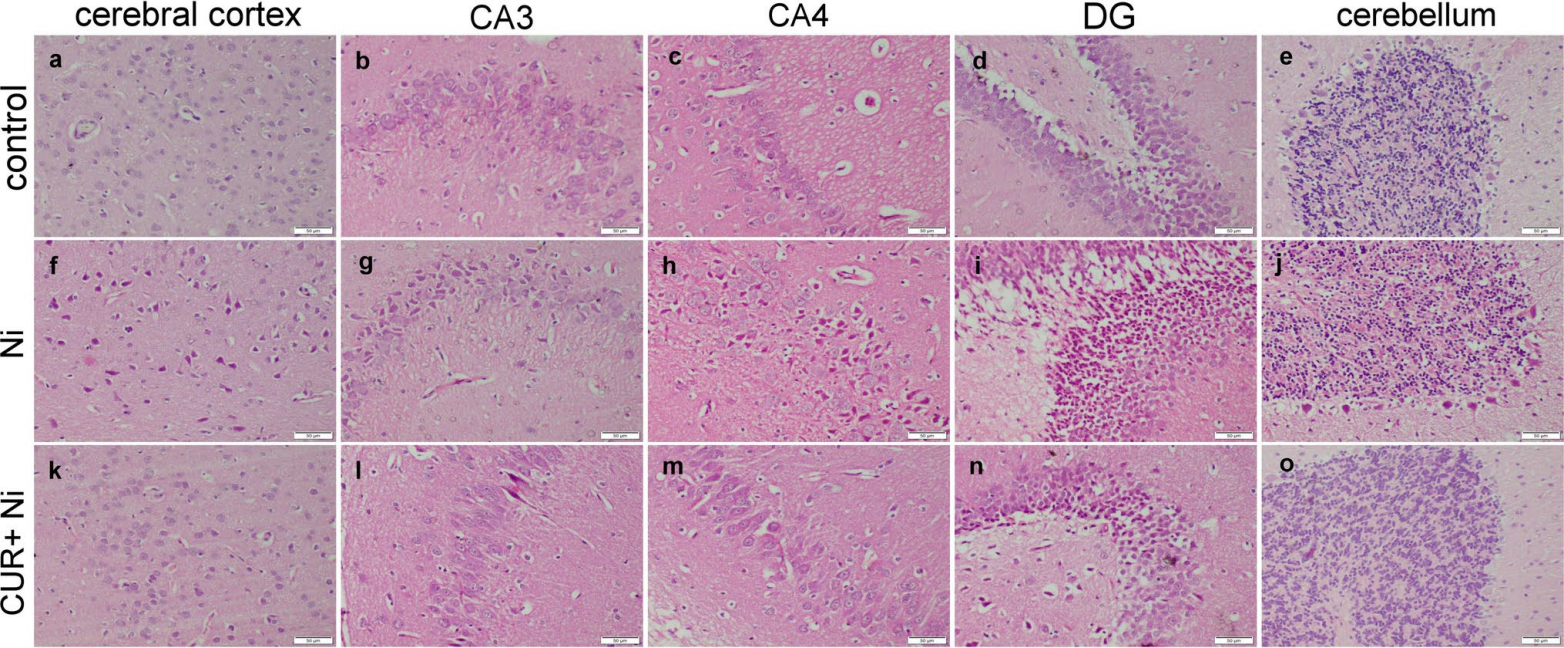
Photomicrograph for histological sections of different brain areas (H&E). Control rat showed histologically normal cerebral cortex (**a**), hippocampus cellular density comprising the CA3 (**b**), CA4 (**c**), dentate gyrus (**d**), and cerebellar molecular, Purkinje cells, and granular layers (**e**). Ni intoxicated rat showed neuronal necrosis with neuronophagia that involves cerebral cortex (**f**), pyramidal neurons of CA3 (**g**) and CA4 (**h**), and granular neuronal cells of dentate gyrus (**i**) and marked loss of Purkinje cells of the cerebellum (**j**). CUR co-treated with Ni rat showed necrosis of individual neurons in the cerebral cortex (**k**), normal cellular density of pyramidal neurons with individual neuronal necrosis comprising the CA3 (**l**) and CA4 (**m**), and mild loss of granular neuronal cells comprising the dentate gyrus (**n**), and individual Purkinje cell necrosis (**o**)

The microscopic examination of different brain areas revealed histologically normal architecture of control and CUR-treated groups. The Ni-intoxicated rats showed the development of neuropathological lesions in different brain areas. Lesions in the cerebral cortex involved neuronal degeneration with neuronophagia with gliosis. The hippocampus showed severe lesions involving all areas extending from CA1 to CA4, and extending into dentate gyrus, the most affected areas were CA3 and dentate gyrus. The lesions were characterized by a reduction of the cellular density of pyramidal neurons comprising CA3 and CA4, in addition to loss of granular cells comprising the dentate gyrus. The cerebellum showed neuronal degeneration in both Purkinje cells. On the other hand, CUR cotreatment markedly improved the microscopic appearance of many brain areas. The cerebrum showed normal histological structure. The hippocampus showed normal histological organization of CA3 and CA4 zones, while DG demonstrated moderate neuronal degeneration. The cerebellum showed individual loss of Purkinje cells, while other parts showed normal histology. The neuropathological lesions in different experimental groups are shown on Fig. 2.

### Immunohistochemical Staining

**Fig. 3:**
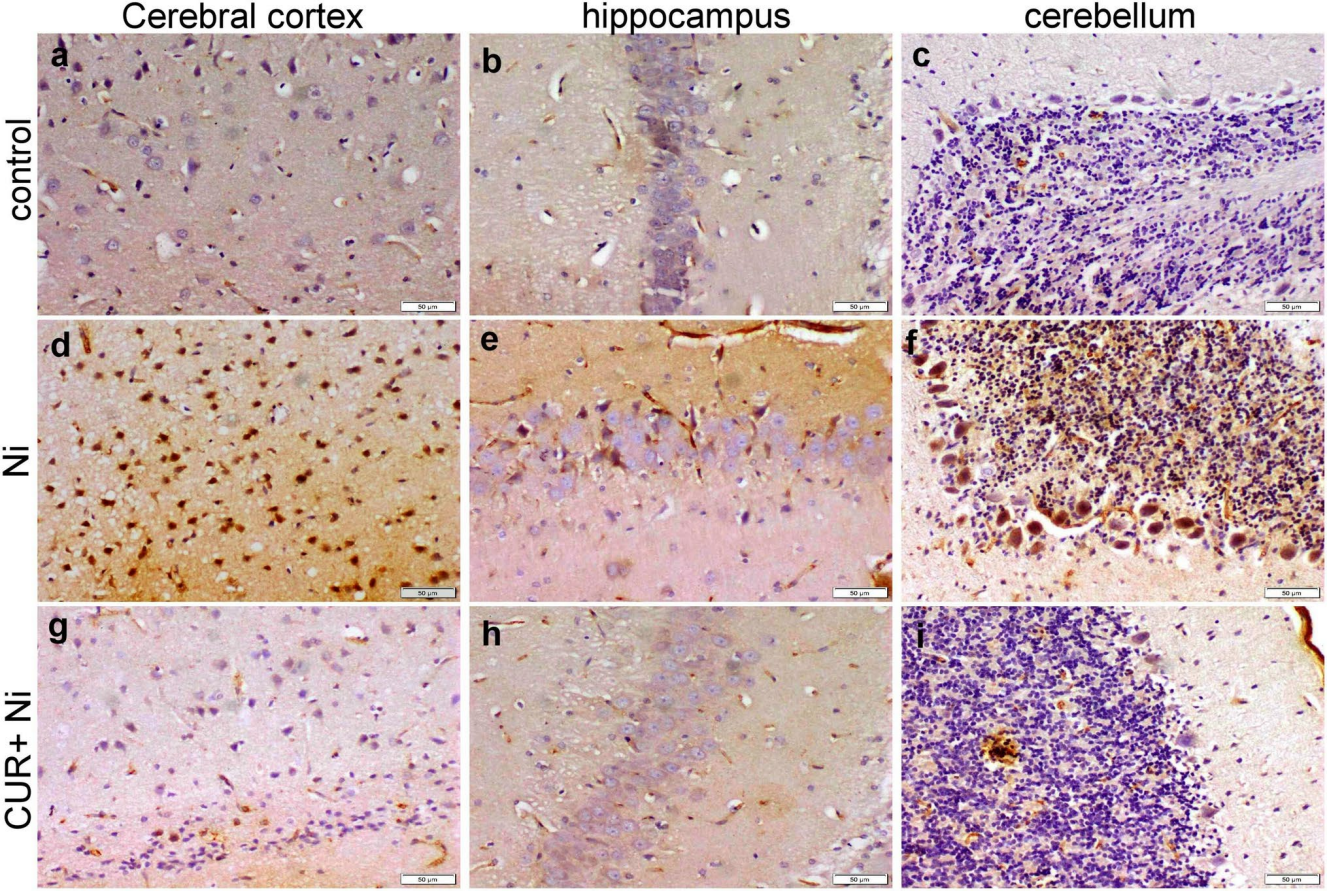
Photomicrograph for histological sections of different brain areas showing the immunoreactivity for Caspase-3. Control rat showed faint weak expression of caspase-3 in the neurons of the cerebral cortex (**a**), hippocampus (**b**), and cerebellum (**c**). Ni intoxicated rats showed strong positive caspase-3 expression in the neurons comprising the cerebral cortex (**d**), pyramidal neurons of the hippocampus (**e**), and Purkinje cells in the cerebellum (**f**). CUR co-treated with Ni rat showed mild caspase-3 expression in neurons in the cerebral cortex (**g**), hippocampus (**h**), and individual Purkinje cells in the cerebellum (**i**)

**Fig. 4:**
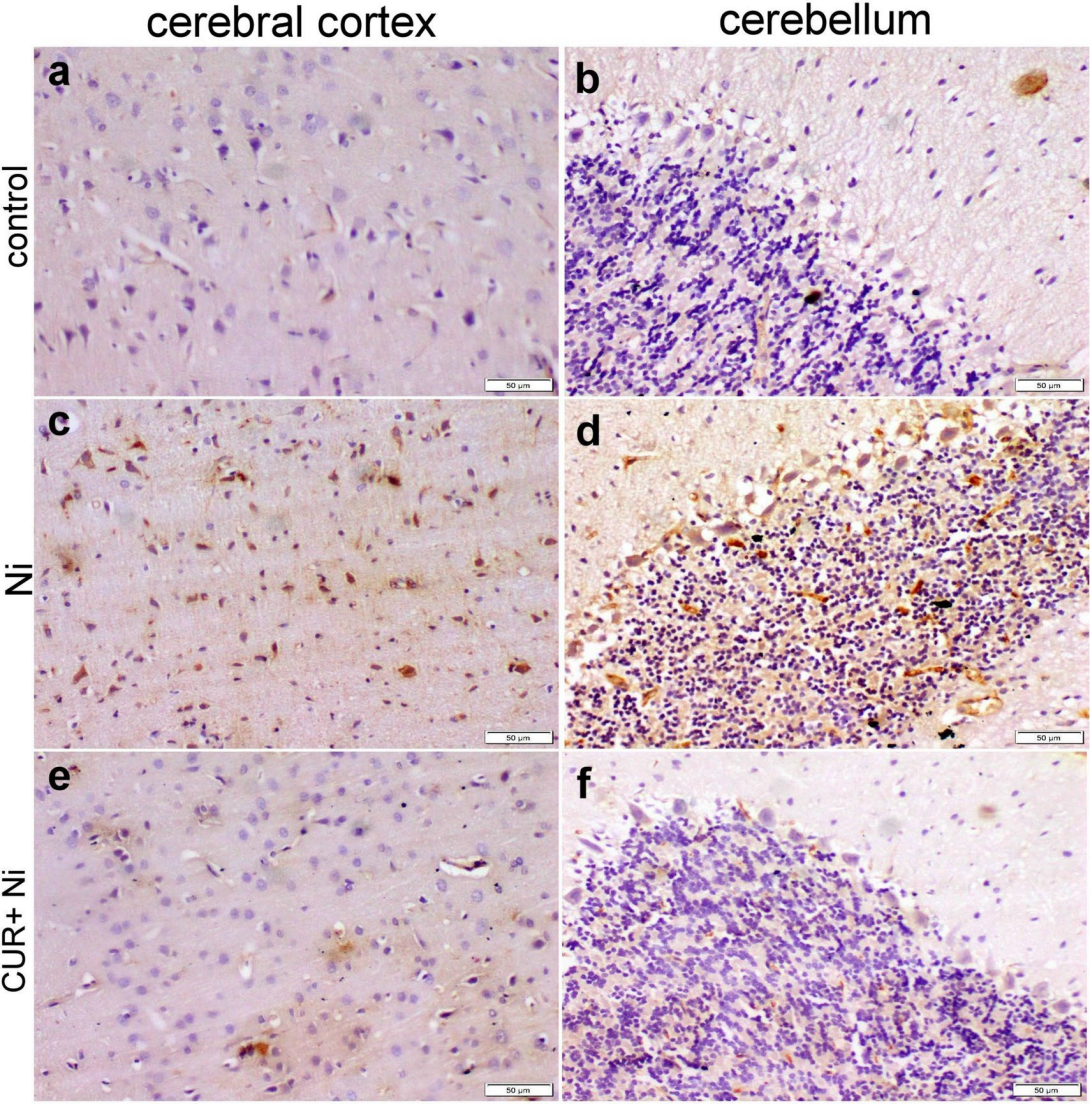
Photomicrograph for histological sections of different brain areas showed the immunoreactivity for NF-κB. Control rat showed negative expression of NF-κB in the neurons of cerebral cortex (**a**) and cerebellum (**b**). Ni intoxicated rat showed strong positive NF-κB expression in the neurons comprising the cerebral cortex (**c**), and Purkinje cells in the cerebellum (**d**). CUR co-treated with Ni rats showed faint NF-κB expression in individual neurons in the cerebral cortex (**e**), and individual Purkinje cells in the cerebellum (**f**)

The immunoreactivity for caspase-3 and NF-κB in different brain areas varied among different groups and was illustrated in Figs. 3 and 4. Strong positive caspase-3 expression in neurons of the cerebral cortex, pyramidal neurons of the hippocampus, and Purkinje cells of the cerebellum were evident, while a marked reduction in expression was detected in CUR cotreated with Ni. Strong positive NF-κB expression in neurons of the cerebral and Purkinje cells of the cerebellum was evident, while a marked reduction in expression was detected in CUR cotreated with Ni.

## Discussion

The present research has been focused on studying the neurotoxic potential of nickel sulfate with related neurobehavioral, biochemical, and pathological alterations with special emphasis on the protective role of curcumin in alleviating nickel intoxication in rats. The neurobehavioral assessment of rats exposed to nickel sulphate and/or curcumin was conducted via three behavioral tests, namely the light/dark transition test, the rod walking test, and the Y-maze test. A light/dark transition test was used in our study to assess the anxiolytic and anxiogenic effects of the different agents [37]. Based on the results of our study, nickel sulphate produced anxiety-like behavior in rats, indicated by more time spent in the dark partition. However, the anxiolytic effect of curcumin was evident since rats exposed to nickel sulphate and curcumin simultaneously spent more time in the light partition compared to the nickel sulphate group. These findings are consistent with those of Yow et al., who showed that curcumin alleviates anxiety-like behavior [38]. In addition, rats’ motor abilities are assessed with the rod walking test. This test is an effective method for identifying locomotor disabilities and determining the effectiveness of agents that affect locomotion and coordination skills, such as tasks requiring coordinated motor and reflex responses [26]. Our results showed that rats exposed to nickel sulphate exhibit impaired motor coordination and balance. The present gait deficits and motor imbalance are related to the developed neuropathological alterations in the cerebellum and mainly affected Purkinje cells, which are responsible for movement, posture, gait, and balance. Ataxic movements are, therefore, one of the clearest signs of cerebellar disorder [39]. In agreement with our findings, nickel sulphate has been shown to cause behavioral anomalies in common carbs, including loss of equilibrium [40]. On the other hand, curcumin improved motor capabilities since there was no significant difference reported between rats treated with nickel sulphate and curcumin and the control rats. Our results agree with those of Spinelli et al., who demonstrated that curcumin enhanced motor activity [41].

Another behavioral test called the Y-maze assay relies on rats’ motivation to investigate new environments. In this test, rats explore a new arm rather than returning to the one they previously investigated [26, 42]. Several brain regions influence this behavior, including the hippocampus, prefrontal cortex, and basal forebrain [43]. Based on our findings, the spontaneous alternation percentage, which measures rats’ natural tendency to alternate between three arms, showed that rats treated with nickel sulphate had poor working spatial memory, as indicated by a decline in their spontaneous alternation percentage. The recorded memory defect and impaired cognition are related to the observed histopathological alterations with neuronal and glial reactions in the hippocampus and primarily affect CA3 and CA4 and dentate gyrus, and this explains the improvement of cognition in rats treated with curcumin as it reduced the grade of neuronal and glial reactions in the hippocampus region. Our results are similar to those of Ijomone and colleagues, who found that nickel sulphate impaired rats'memory and learning as well [2]. Zhang et al., demonstrated that curcumin enhanced cognitive function and recognition memory, which is consistent with our findings [20].

The brain is commonly recognized as a significant target area in Ni-induced neurotoxicity. After exposure, Ni majorly accumulates in the cerebral cortex, where it induces neurological damage [6, 44]. Ni neurotoxic effects have been linked to a variety of pathways. Among these pathways include disruptions in energy metabolism, oxidative stress, inflammation, genetic alteration, and apoptosis [2]. The present study proved the disruption of the redox state-mediated neurotoxic potential of Ni, as a significant disruption in the brain redox status manifested with elevation of lipid peroxidation (MDA) and nitrosative stress (NO) as well as depletion of glutathione content in the group received Ni only and markedly ameliorated after curcumin treatment, previous results reported significant inhibition of antioxidant enzymes like superoxide dismutase, catalase, and glutathione reductase due to exposure to Ni in rats [45]. In addition, the AChE is activated to hydrolyze acetylcholine, an essential neurotransmitter, resulting in the termination of the synaptic transmission. Thus, the significant inhibition of the brain AChE activity in rats exposed to Ni alone in this study is associated with oxidative damage due to dysregulation of brain oxidative balance.

The disruption in the redox state in brain tissue is correlated with the neuronal degeneration that was detected in different brain regions mainly the cerebral cortex, hippocampus, and cerebellar Purkinje cells with marked immunoreactivity of caspase 3 and NF-κB, the role of increased cellular redox state in activation of NF-κB and apoptotic pathways was confirmed as IKK-based NF-κB-inducing signaling process is activated by increased cellular oxidative stress [46]. Moreover, the significant reduction in the transcriptase levels of Nrf2 and HO-1 genes in the brain tissue of the group that received Ni reflects the oxidative stress induction by Ni as Nrf2 regulates antioxidant enzymes that share in the detoxification and elimination of oxidative [47, 48], Nrf2 increases superoxide dismutase and is the most important regulator of HO-1 [49]. Interestingly, the current study revealed that the co-treatment of curcumin with Ni significantly improved brain redox status via reduction of NO and MDA and GSH restoration, as well as significant enhancement in the brain AChE activity. In accordance with previous studies, curcumin may be a useful antioxidant that lessens oxidative stress’s negative consequences. It lowers oxidative stress by interacting with several molecular pathways, which is linked to its capacity to chelate heavy metals and control the activity of numerous enzymes [50–52].

## Conclusion

The present data emphasize possible pathways that mediate the nickel sulfate neurotoxicity and correlate the neurobehavioral abnormalities with selective targeting of brain areas for neuropathology progression, disruption of the redox state in the brain regions, and downregulation of NrF2 and HO-1 expressions. The role of curcumin in preventing Ni-induced intoxication was confirmed via antioxidant and anti-apoptotic pathways and downregulation of NF-κB in the brain.

## Data Availability

No datasets were generated or analysed during the current study.
